# Failed Orthodontic PEEK Retainer: A Scanning Electron Microscopy Analysis and a Possible Failure Mechanism in a Case Report

**DOI:** 10.3390/dj12070223

**Published:** 2024-07-18

**Authors:** Piero Antonio Zecca, Salvatore Bocchieri, Andrea Carganico, Margherita Caccia, Rosamaria Fastuca, Marina Borgese, Luca Levrini, Marcella Reguzzoni

**Affiliations:** 1 Department of Medicine and Innovative Technologies, University of Insubria, 21100 Varese, Italy; pieroantonio.zecca@uninsubria.it (P.A.Z.);; 2 Department of Biomedical and Dental Sciences, Morphological and Functional Images, University of Messina, 98122 Messina, Italy; salvatore.bocchieri@unime.it; 3 Department of Biotechnology and Life Sciences, University of Insubria, 21100 Varese, Italy; 4 Private Practice, 6900 Lugano, Switzerland; 5 Department of Human Science and Innovation for the Territory, University of Insubria, 21100 Varese, Italy

**Keywords:** polyetheretherketone, orthodontic retainer, scanning electron microscopy SEM, orthodontic relapse

## Abstract

This study presents a scanning electron microscopy analysis of a failed PEEK retainer in an orthodontic patient. After 15 months of use, the patient reported a gap opening between teeth 41 and 42. The PEEK retainer was removed and sent for electron microscope analysis. To investigate the failure, scanning electron microscopy was employed to assess the microstructure and composition of the retainer at various magnifications. These findings suggest that the failure of the PEEK retainer was multifaceted, implicating factors such as material defects, manufacturing flaws, inadequate design, environmental factors, and patient-related factors. In conclusion, this scanning electron microscopy analysis offers valuable insights into the failure mechanisms of PEEK retainers in orthodontic applications. Further research is necessary to explore preventive strategies and optimize the design and fabrication of PEEK retainers, minimizing the occurrence of failures in orthodontic practice.

## 1. Introduction

Orthodontic retainers play a crucial role in maintaining the achieved alignment and stability of teeth following orthodontic treatment. They provide long-term support to prevent the teeth from relapsing into their original positions, ensuring the success and longevity of orthodontic outcomes [1]. Due to dental physiology, teeth tend to shift towards the middle over time. Following orthodontic therapy, the essential use of a retainer is imperative. This prevents regression to the original misalignment and guards against potential worsening of the condition [2,3,4].

Braided stainless steel wire is usually the recommended material for retainers’ post-orthodontic treatment. To secure the teeth in their corrected placements, this wire, which is thin and flat by nature, must be positioned strategically from canine to canine along the lingual portion of the lower dental teeth. This wire needs to be placed precisely, giving particular attention to every detail. The wire must be positioned passively and precisely to follow the curvature of the dental parts’ lingual surfaces. Insufficient adjustment of the wire could cause unintended movements of the teeth, which could compromise the orthodontic result [5,6,7,8].

To create a retainer, long metal ligatures that are manually braided can also be used. However, although they adapt well to the shape of the tooth and are comfortable, they are highly deformable, easily detach, and do not withstand the forces acting in the oral cavity well. Prefabricated metal bars with two adhesive plates only on the canines can also be an alternative. However, these are not adhered to at the level of the incisors, and, therefore, while they provide good maneuverability for oral hygiene, they do not prevent physiological movements [9].

Fiber-reinforced materials, such as fiberglass retainers, are also used next to steel wires. These materials are made from layers of fibers, which can be glass, carbon, polyamide, and polyethylene, separated and surrounded by resin. Because of their color, which is similar to that of natural teeth, these materials offer a better aesthetic appearance. However, they lose their durability over time, and the link between the composite material and the tooth is not as strong as it is with metal [5,6,7,8]. These materials significantly solidify the anterior group, and they have a high degree of rigidity, which results in a poor stretching capacity and contributes to the detachment in addition to the adhesive contact. As a result, the material undergoes a lot of stress, which leads it to split [8].

Among the various materials used for retainer fabrication, polyetheretherketone (PEEK) has emerged as a promising alternative to traditional materials, such as stainless steel and acrylic. In fact, in the UK in 1998, PEEK was suggested as a suitable material for use in biomedical applications. PEEK founded its application in maxillofacial surgery during bone substitution, orthopedic surgery, neurosurgery, cardiac surgery, and dentistry [10].

PEEK, a high-performance thermoplastic polymer with a polyaromatic structure, offers numerous advantages, including biocompatibility, aesthetics, and ease of customization [11]. PEEK has gained popularity for medical and dental applications [10,12,13,14,15] due to its excellent mechanical properties, which include high strength, resilience, elasticity, and flexibility. The flexibility module of PEEK is 140–170 MPa and the elasticity module is 3–4 GPa. These values are closer to enamel and dentin compared to multistranded stainless steel wires.

These properties allow PEEK to be used for orthodontic retainers to withstand the forces exerted by the surrounding oral environment, including mastication and tongue movement, while maintaining their shape and functionality. Moreover, PEEK exhibits favorable biocompatibility, reducing the risk of allergic reactions and soft tissue irritation commonly associated with other retainer materials, making it a suitable material for use in the human body [13]. So, thanks to its hypoallergenic properties, PEEK can also be used in individuals allergic to metals, such as titanium.

In addition to its mechanical and biocompatible properties, PEEK retainers provide enhanced aesthetics compared to traditional metallic retainers [14]. Their translucent nature allows for a more discreet appearance, reducing patient self-consciousness during retainer wear. This aesthetic advantage is particularly important for adolescent and adult patients who desire a more cosmetically appealing option.

Additionally, PEEK is a material that retains less plaque compared to metal. This is a significant advantage, considering the multitude of studies highlighting how the application of a retainer is then associated with periodontal problems caused by substantial plaque accumulation and the challenges in cleaning it [5,10,16,17].

PEEK can also be sterilized because its mechanical properties do not change during the process due to its thermal stability up to 335.8 °C [18].

It is commonly used for orthodontic retainers and other orthodontic devices [19].

Customization is a critical aspect of orthodontic retainers, as each patient’s dental anatomy is unique. PEEK retainers can be fabricated using computer-aided design and computer-aided manufacturing (CAD/CAM) technology, enabling precise adaptation to individual dental arches. This customization enhances patient comfort and ensures optimal fit, leading to improved patient compliance and satisfaction. Despite the advantages offered by PEEK retainers, it is important to acknowledge that failures and complications can occur with any dental appliance. Understanding the factors that contribute to the failure of PEEK retainers is crucial for their continued development and refinement. Investigating failure cases can provide insights into material weaknesses, manufacturing flaws, design limitations, and patient-related factors that may compromise the performance and longevity of PEEK retainers.

This case report presents the failure of a polyetheretherketone (PEEK) dental retainer in a patient undergoing orthodontic treatment. Notwithstanding its promising mechanical and biological properties, limited data are available on the clinical performance and durability of PEEK retainers [20]. This study aims to investigate the potential limitations and failure process of PEEK retainers and raise awareness among clinicians and researchers in order to improve the material and the potential field of application. In the experience of the patients treated by the authors, the failure rate of peek retainers is 66% (regarding debonding, relapse, space opening, and unwanted orthodontic movement). In this background, it is crucial to develop and investigate new technologies and techniques in the field. Scanning electron microscopy analysis could represent an outstanding tool to investigate microscopic and morphological aspects of dental materials surfaces. A limitation of this paper is the analysis of a single case based on an experimental technique.

## 2. Materials and Methods

**Figure 1:**
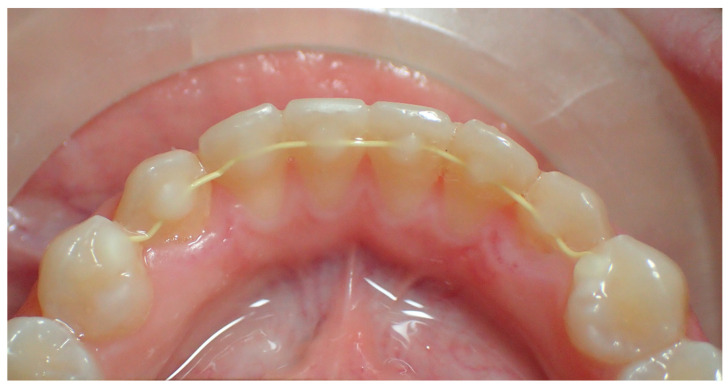
Final photo with retainer applied.

The 25-year-old female patient (with no relevant medical and dental anamnesis) in this case report underwent orthodontic treatment and required a PEEK retainer for periodic MRI exams (Figure 1). A post-treatment intraoral 3D scan was taken. The retainer used in this case report was produced using Ossfila Medical PEEK Filament (round shaped 1.2 mm of the section) printed with 333 DRY (3d Dream, Cassano Magnago, Italy), a type of PEEK polymer specifically formulated for medical and dental applications. Three-dimensional printed technology in orthodontics has created customized and special appliances, including retainers.

The printed retainer was bonded according to Beretta et al.’s suggestions [11]. The PEEK retainer was tested on the lingual surface of the lower anterior teeth before the bonding procedures to verify its passivity and adaptation, which have to be perfect. Once this was ensured, the retainer was roughened with a diamond bur and then a silane agent was applied on the surface. This agent was allowed to evaporate for 30 s and, after that, it was cured for again 30 s. This initial step prepares the PEEK surface for adhesion.

Etching (37% orthophosphoric acid) was applied to the lingual areas of the lower anterior teeth. After 30 s, it was rinsed off, and the surface was carefully dried. An adhesive layer was applied to the same surface, taking care not to interest the interproximal surface. After curing it for 20 s, the PEEK retainer was held in place with dental floss to ensure correct positioning. The dental floss is threaded between the interdental contact points of the involved teeth, creating a loop. The retainer is then passed through these loops, which are subsequently tightened. This secures the wire in place and allows for verification and adjustment of its position.

Then, composite resin was applied over the retainer and on the lingual surface of the involved teeth and the retainer, except at the contact points, and it was cured carefully. After the curing phase, the dental floss is removed, and any excess composite is cleaned off. It is also very important to verify that enough space has been left to allow the passage of the ortho-floss [11].

**Figure 2:**
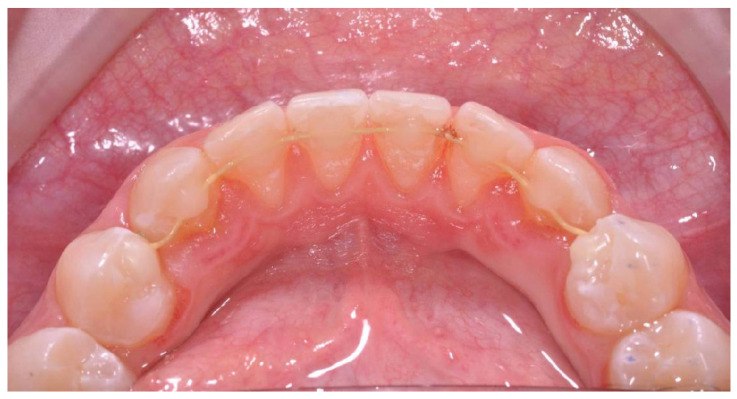
Photo after 15 months; note the opening of the space between teeth 41 and 42 due to orthodontic relapse.

After 15 months of use, this patient reported experiencing a slight problem with the retainer, evidenced by a gap between teeth 41 and 42 (Figure 2).

To understand the cause of this failure without any detachment of the composite, the retainer was removed and sent to the Human Morphology Laboratory of the University of Insubria for scanning electron microscopy analysis. The specimens were then thawed in Karnovski’s solution and dehydrated in graded ethanol and hexamethyldisilazane. All specimens were mounted on appropriate stubs with a colloidal silver glue, gold coated in an Emitech K550 sputter coater (Emitech, Beaucouzé, France), and observed with an FEI XL-30 FEG high-resolution SEM (now Thermo Fisher, Waltham, MA, USA). Images were directly obtained as 8 bpp, 1424 × 968 TIFF files [21]. To control for material differences and ensure accurate results, a new PEEK retainer that had never been applied was also analyzed at the same time as the failed retainer.

## 3. Results

**Figure 3:**
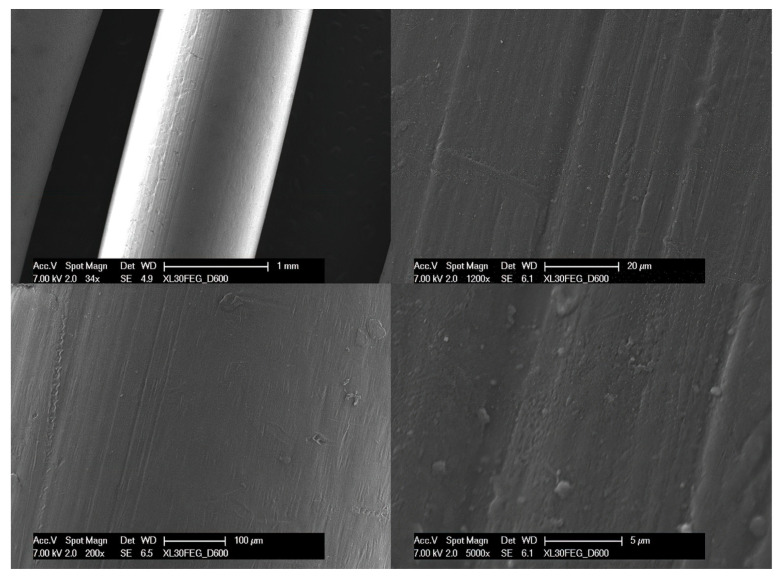
Scanning electron microscopy images; note the smooth and compact surface of the material.

As demonstrated by the scanning electron microscopy images, the control specimen (new retainer) had a smooth surface, making it difficult for bacteria to adhere to and contaminate it (Figure 3). This observation highlights the importance of material surface properties in the long-term performance and durability of orthodontic appliances, including PEEK retainers. Comparing the control specimen and the failed retainer will provide essential insights into the morphological and structural changes that occur over time and will contribute to the ongoing evaluation of the clinical performance and durability of PEEK retainers.

**Figure 4:**
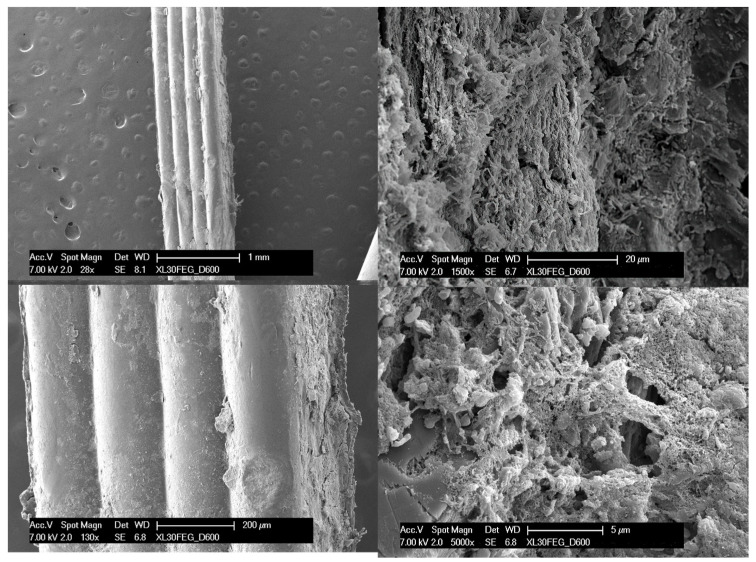
Scanning electron microscopy image; note how the material begins to delaminate and there is widespread bacterial contamination.

**Figure 5:**
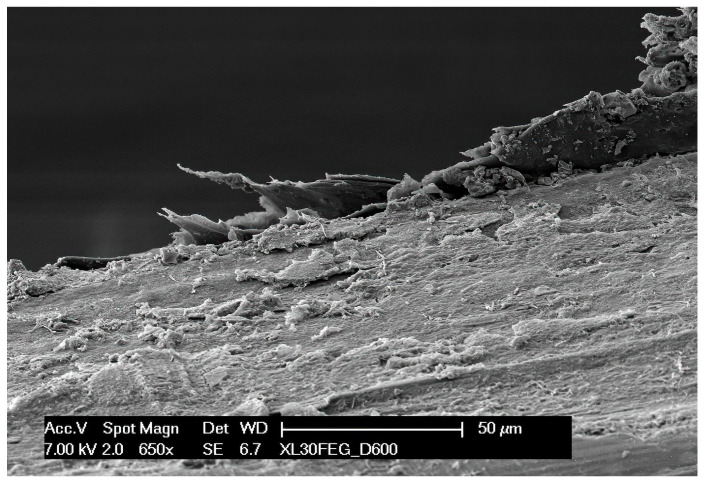
Delamination detail.

In the failed retainer, a loss of surface continuity (likely the cause of the opening of the interdental space between teeth 41 and 42) and a strong adherence of bacterial colonization to the surface can be observed through electron microscopy images (Figure 4). A delamination in the surface was easily found, allowing bacteria adhesion (Figure 5).

## 4. Discussion

The findings of this case report will be of significant value to orthodontic practitioners in their decision making regarding using PEEK retainers in post-treatment retention. These findings suggest that the material properties of PEEK retainers, including surface roughness and hygroscopicity, play a critical role in their long-term clinical performance and durability. The results of this case report highlight the importance of carefully monitoring patients who receive PEEK retainers and considering alternative post-treatment retention options for those at higher risk for retainer failure. The findings of this case report will contribute to the ongoing evaluation of PEEK retainers and provide valuable information for orthodontic practitioners in their decision making regarding their use. The results indicate that the delamination of the PEEK retainer creates a situation where bacteria can colonize the retainer in depth. This can occur when the surface of the retainer becomes rough and porous, creating an environment conducive to the growth of bacteria. The electron microscopy images show that the delamination of the retainer has resulted in a loss of surface continuity, which has allowed bacteria to penetrate the material and establish a strong adherence to the surface. When examining the failure of an orthodontic retainer made from PEEK (polyetheretherketone), it is essential to understand how this material interacts with saliva over extended periods. PEEK is a high-performance thermoplastic renowned for its excellent mechanical properties and chemical resistance. However, it does have a low level of water absorption, typically around 0.1% to 0.5% when fully saturated.

Although PEEK’s water absorption rate is relatively low, continuous exposure to the moist environment of the mouth, where the retainer is in constant contact with saliva, can lead to subtle but cumulative effects. Over time, the absorbed water acts as a plasticizer, which lowers the glass transition temperature and increases the ductility of the material. This process can result in a slight increase in the volume of the retainer, manifesting as swelling.

This swelling might seem minor, but in the precise fit required for orthodontic retainers, even small changes can alter the effectiveness of the device. If the retainer swells, it might not hold the teeth in the correct position, leading to a failure in its function. Moreover, the continuous cycle of wetting and drying, as the retainer is worn and then removed, could exacerbate these changes, potentially leading to material fatigue or micro-cracks over time.

While PEEK is resistant to hydrolytic degradation, its slow rate of water absorption and the effects of an aqueous environment, like saliva, can compromise the structural integrity and performance of an orthodontic retainer. Thus, the failure of a PEEK retainer could be attributed to these gradual changes in the material properties due to prolonged exposure to a moist environment. Water absorption is a crucial factor to consider when using PEEK, as the wire is very thin and immersed in a constantly damp environment, like the oral cavity. The fibers that make up the retainer wire are few and had a smaller diameter compared to other medical structures made of PEEK, such as hip or spinal prostheses. Consequently, the amount of water needed to separate the fibers of the wire is much lower than that required to separate the fibers of larger structures, making the PEEK wire more exposed to breakage.

Another parameter to consider when choosing PEEK material is the elongation at the break, which is independent of hydrophilicity. Steel wires, when subjected to tensile force, do not deform but resist until they reach the breaking point, which varies depending on the size of the wire. In contrast, PEEK wires elongate by 40–50% when subjected to tensile force at a temperature of 23 °C, the typical temperature of the oral cavity. Only after reaching this elongation do PEEK wires undergo a breaking mechanism. This is important to consider because in the case of parafunction, PEEK undergoes constant deformation, unlike steel, which maintains its shape.

This comparison will provide a reference point for the SEM analysis, allowing for a more comprehensive evaluation of the morphological and structural changes that occurred in the failed retainer. Comparing the two retainers will provide valuable insights into the potential limitations and failure mechanisms of PEEK retainers and contribute to the ongoing evaluation of their long-term clinical performance. This highlights the importance of carefully monitoring patients receiving PEEK retainers and the need to consider alternative post-treatment retention options for those at higher risk for retainer failure. The results of this case report provide valuable insights for orthodontic practitioners and researchers in understanding and developing the material properties in the long-term clinical performance and durability of PEEK retainers [22,23,24].

## 5. Conclusions

In conclusion, based on this case report, it is currently impossible to make a clinical recommendation for using PEEK retainers in all patients, as there is a risk of retainer failure. The discontinuity observed in this case may be due to the minimal hygroscopicity of the PEEK material, which tends to absorb water and change the surface. The author believes that PEEK retainers may be useful in patients who require repeated magnetic resonance imaging in adjacent areas. Still, they must be kept under constant orthodontic monitoring to avoid recurrence problems. More studies on a wide sample are necessary in order to have more comparative data. Further research is needed to fully understand the clinical significance of PEEK retainers, improve their mechanical and biological characteristics with any kind of surface treatment, and establish guidelines for their safe and effective use in orthodontic treatment.

## Data Availability

Data are available from the corresponding author upon reasonable request.
